# A case report of delayed cortical infarction adjacent to sulcal clots after traumatic subarachnoid hemorrhage in the absence of proximal vasospasm

**DOI:** 10.1186/s12883-018-1217-y

**Published:** 2018-12-18

**Authors:** Christian Schinke, Viktor Horst, Ludwig Schlemm, Matthias Wawra, Michael Scheel, Jed A. Hartings, Jens P. Dreier

**Affiliations:** 1 Department of Neurology, Charité - Universitätsmedizin Berlin, Freie Universität Berlin, Humboldt-Universität zu Berlin, Berlin Institute of Health, Berlin, Germany; 2 Center for Stroke Research Berlin, Charité - Universitätsmedizin Berlin, Freie Universität Berlin, Humboldt-Universität zu Berlin, Berlin Institute of Health, Berlin, Germany; 3 Department of Experimental Neurology, Charité - Universitätsmedizin Berlin, Freie Universität Berlin, Humboldt-Universität zu Berlin, Berlin Institute of Health, Berlin, Germany; 4 Berlin Institute of Health (BIH), Berlin, Germany; 5 London School of Economics and Political Science, London, UK; 6 Department of Neuroradiology, Charité - Universitätsmedizin Berlin, Freie Universität Berlin, Humboldt-Universität zu Berlin, Berlin Institute of Health, Berlin, Germany; 7 Department of Neurosurgery, University of Cincinnati (UC) College of Medicine, Cincinnati, OH USA; 8 Bernstein Center for Computational Neuroscience Berlin, Berlin, Germany; 9 Einstein Center for Neurosciences Berlin, Berlin, Germany

**Keywords:** Traumatic subarachnoid hemorrhage, Delayed ischemic neurological deficits, Cortical spreading depolarization

## Abstract

**Background:**

Cortical ischemic lesions represent the predominant pathomorphological pattern of focal lesions after aneurysmal subarachnoid hemorrhage (aSAH). Autopsy studies suggest that they occur adjacent to subarachnoid blood and are related to spasm of small cortical rather than proximal arteries. Recent clinical monitoring studies showed that cortical spreading depolarizations, which induce cortical arterial spasms, are involved in lesion development. If subarachnoid blood induces adjacent cortical lesions, it would be expected that (i) they also develop after traumatic subarachnoid hemorrhage (tSAH), and (ii) lesions after tSAH can occur in absence of angiographic vasospasm, as was found for aSAH.

**Case presentation:**

An 86-year-old woman was admitted to our hospital with fluctuating consciousness after hitting her head during a fall. The initial computed tomography (CT) was significant for tSAH in cortical sulci. On day 8, the patient experienced a secondary neurological deterioration with reduced consciousness and global aphasia. Whereas the CT scan on day 9 was still unremarkable, magnetic resonance imaging (MRI) on day 10 revealed new cortical laminar infarcts adjacent to sulcal blood clots. Proximal vasospasm was ruled out using MR and CT angiography and Doppler sonography. CT on day 14 confirmed the delayed infarcts.

**Conclusions:**

We describe a case of delayed cortical infarcts around sulcal blood clots after tSAH in the absence of proximal vasospasm, similar to results found previously for aSAH. As for aSAH, this case suggests that assessment of angiographic vasospasm is not sufficient to screen for risk of delayed infarcts after tSAH. Electrocorticography is suggested as a complementary method to monitor the hypothesized mechanism of spreading depolarizations.

## Background

In autopsy studies, cortical ischemic lesions are the predominant pattern of parenchymal damage both in patients with aneurysmal subarachnoid hemorrhage (aSAH) [1, 2] and in the non-human primate model of aSAH [3]. The cortical lesions typically occur adjacent to subarachnoid blood clots. They showed no relationship with angiographic vasospasm of the large cerebral arteries in either humans or non-human primates [1–3]. It has been suggested that the most likely explanation for these infarcts is vasospasm of cortical resistance arteries and arterioles caused by blood products [2]. Vasospasm in these small vessels cannot be resolved using digital subtraction angiography (DSA).

The autopsy observations are consistent with the clinical finding that thick layers of subarachnoid blood on admission computed tomography (CT) scans are among the most important predictors of infarction and unfavorable outcome after aSAH [4]. If adjacent cortical lesions are induced by subarachnoid blood, it would be expected that (i) they also develop after traumatic subarachnoid hemorrhage (tSAH), and (ii) lesions after tSAH can occur in absence of angiographic vasospasm, as was found for aSAH [5–8]. In fact, both early and delayed ischemic lesions have been described after tSAH [9]. However, to our knowledge, it has not been documented that delayed lesions can develop without angiographic vasospasm. Here, we report a case of tSAH in which delayed cortical infarcts developed around sulcal blood clots in the absence of proximal vasospasm.

## Case presentation

An 86-year-old woman was admitted from an outside institution to our neurological intensive care unit with fluctuating consciousness after hitting her head during a fall. Four weeks before admission, she was in normal health for her age with a history of arterial hypertension. Because of atrial fibrillation, she was treated with rivaroxaban 20 mg once daily. Two weeks before hospitalization, she experienced pain in her lower abdomen accompanied by a feeling of illness and fatigue which she self-medicated with aspirin. She experienced nose bleeding but continued to take aspirin. She was then admitted to an external clinic after falling. During the first in-hospital night, she fell out of bed and struck her head. Thereafter, consciousness decreased and she was transferred to the neurocritical care unit of our institution. She presented with dysarthria and mild motor aphasia, but language comprehension was fully preserved. In addition, mild right-sided hemiparesis was noted. Most of the time, she was awake but intermittently somnolent. Body temperature was 37.6 °C. Routine laboratory tests revealed prolonged prothrombin time, increased international normalized ratio (INR), increased CRP, leukocytosis and corresponding signs of a urinary tract infection. She was treated with prothrombin complex concentrate and antibiotics.

**Fig. 1:**
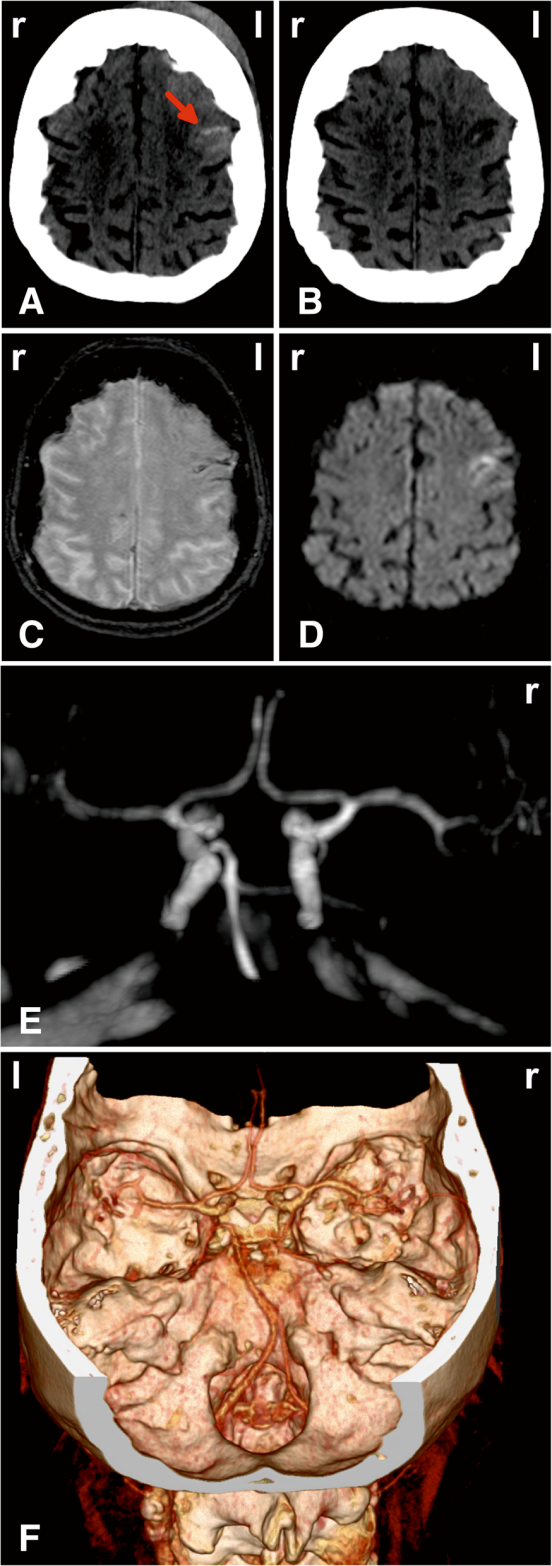
Development of a cortical infarct around sulci filled with subarachnoid blood. (**a**) is a representative image of the initial CT scan on day 0. It shows subarachnoid blood in two sulci of the left frontal cortex (arrow). In addition, a left-sided hematoma exterior of the head marks the area of the impact. (**b**) Whereas the subsequent CT scans on days 1, 3, 5 and 9 showed no new infarcts, the follow-up CT scan on day 14 revealed a new hypodensity confined to the cortical gray matter around the sulci that had shown subarachnoid blood in the CT scan on day 0. This lesion was consistent with the new infarct seen on the MRI scan of day 10. (**c**) The T2* weighted image of the MRI on day 10 revealed that the subarachnoid blood was still present in the sulci in which it had been seen on the initial CT scan, although the hyperdensity had disappeared on later CT scans. (**d**) The DWI on day 10 showed a hyperintensity typical of a new infarct around the sulci with subarachnoid blood. This DWI hyperintensity corresponded to an ADC reduction (not shown). The synopsis of images (**a**) – (**d**) provides evidence for delayed cortical laminar necrosis adjacent to the sulcal clot in the left frontal cortex. (**e**) The MRA on day 10 and (**f**) the CTA on day 14 did not show any evidence of proximal vasospasm

The initial computed tomography (CT) on day 0 showed contusions in the left frontal and temporal lobes and tSAH. Figure 1a shows this first CT scan with subarachnoid blood in two sulci of the left frontal cortex. A contre-coup injury was found in the right posterior cranial fossa with an epidural hematoma and corresponding tSAH. In addition, a small intra-parenchymal hemorrhage was observed in the right basal ganglia. Arterial aneurysms or arteriovenous malformation were ruled out using CT angiography (CTA). Blood was also detected in the fourth ventricle, but signs of disturbed cerebrospinal fluid circulation were not seen. Accordingly, the patient did not receive external ventricular drainage. Further CT scans on days 1, 3 and 5 showed neither increase of the epidural hematoma nor development of an occlusive hydrocephalus. They revealed only the progressive decrease of Hounsfield units within the blood-related hyperdensities. Nimodipine was not given because there is currently no recommendation for its use after tSAH [9].

The patient was alert and in an improved general condition when she was transferred to the normal ward on day 7. On day 8, however, the patient experienced a secondary deterioration of her neurological status, as characterized by reduced consciousness and global aphasia, with loss of ability to speak or understand. Whereas the CT scan on day 9 was still unremarkable, the MRI scan on day 10 revealed new cortical laminar infarcts adjacent to the sulcal clots (Fig. 1 c and d). The infarcts included both Broca and Wernicke areas. Transcranial Doppler sonography (TCD) on day 10 showed normal mean velocities of the posterior cerebral arteries (right PCA 29 cm/s, left PCA 23 cm/s) and normal pulsatility indices. The bone window was not sufficient to assess velocities of the middle cerebral arteries (MCA). Neither MR angiography (MRA) on day 10 nor CTA on day 14 showed any signs of proximal vasospasm (Fig.1 e and f). However, the CT scan on day 14 revealed the same infarcts as the MRI scan on day 10 (Fig. 1 b). Over the following week, the patient improved again. She was able to respond to simple commands when she was transferred to clinical rehabilitation.

## Discussion and conclusions

In addition to contusions and an epidural hematoma, this patient experienced tSAH with sulcal blood clots and initial focal neurological deficits. After initial improvement, this was succeeded by a secondary neurological deterioration with global aphasia on day 8. Accordingly, an MRI on day 10 showed new infarcts around sulcal blood clots. In contrast to the CT scan on day 9, the CT scan on day 14 also showed the new infarcts. No signs of proximal vasospasm were found using MRA, CTA and TCD.

A limitation of the study is that, despite serial use of multiple diagnostic procedures, there is a theoretical possibility that proximal vasospasm occurred but escaped detection. For example, the bone windows for TCD examination were sufficient to assess the PCAs but not the MCAs. It is also possible that proximal vasospasm occurred in connection with new infarct development between days 9 and 10 but resolved rapidly and was not detected by MRA on day 10. However, this scenario is unlikely since proximal vasospasm after aSAH, as assessed by either TCD or DSA, typically resolves only after day 16 [10, 11]. Another consideration is that while CTA and MRA are reliable noninvasive methods to assess proximal diameters of the basal cerebral arteries, they are inferior to DSA for evaluation of the more distal arterial branches [12, 13]. Accordingly, we cannot exclude that vasospasm occurred in these more distal branches in our patient. Nonetheless, several lines of evidence suggest that cortical ischemic lesions after aSAH are primarily the consequence of spasm in the cortical microvessels that lie beyond the resolution of DSA, as explained below [14, 15].

Cortical ischemic lesions similar to those reported here were observed by Stoltenburg-Didinger and Schwarz in 106 of 139 (76%) autopsy cases of ruptured aneurysms [2]. Notably, intravascular thrombi occurred in only 4 of the 106 autopsy cases with infarcts, and it was concluded that these thrombi did not precipitate the infarcts, but rather resulted from microcirculatory disorders secondary to the developing necrosis. Endothelial swelling was also excluded as an etiologic factor since this occurs temporarily and would only obstruct the lumina of capillaries, not arteries or arterioles. Compression was unlikely as a potential cause since subarachnoid clots would lead to venous prior to arterial compression due to the thinner vessel wall of veins. Venous compression would result in primarily hemorrhagic infarcts, but the cortical infarcts were always anemic. Finally, no relationship was found between the cortical lesions and angiographic vasospasm of the large cerebral arteries in either humans or non-human primates [1–3]. The only consistent finding in the autopsies of both patients and monkeys was that the cortical lesions typically occurred adjacent to subarachnoid blood clots, suggesting that blood products are involved in their pathogenesis [2, 3]. In the pathoanatomical descriptions, the cortical infarcts were of different shape and size. Most of them were bell-shaped, corresponding to the territory of small perforating arteries, or laminar, corresponding to the territories of rectangular branches of cortical arteries. It was suggested that the most likely explanation for these infarcts is spasm of the cortical arteries [2].

The first experimental evidence that subarachnoid blood clots can cause focal cortical necrosis was provided almost 70 years ago by Iwanowski and Olszewski, who published a study on ‘subpial cerebral siderosis’ [16]. After injections of blood into the subarachnoid space, the dogs in this study developed neurological deficits on the following days, and pathologic changes included the occurrence of cortical laminar necrosis. A similar result was recently found in the swine sulcal clot model, in which subarachnoid blood alone was sufficient to cause adjacent cortical infarcts [17]. Notably, their development was associated with spreading depolarizations (SD), in agreement with previous rat studies showing that subarachnoid blood products induce SDs. A key aspect of SDs in the presence of blood products is that they induce an inverse neurovascular response, known as spreading ischemia, in contrast to the arteriolar dilation and spreading hyperemia observed in response to SD in normal cortex [18, 19]. Thus, SDs in the presence of blood products initiated severe long-lasting spasm of cortical arteries with consequent spreading ischemia. Spreading ischemia alone was sufficient to cause cortical infarcts that have similar pathologic morphology as observed in patients with aSAH. Subsequently, SDs and spreading ischemias were described in patients with aSAH [20] and also those with tSAH [21]. Thus, in the wake of both aSAH and tSAH, inverse neurovascular coupling in response to SDs might acutely exacerbate the microarterial spasm resulting from cortical exposure to blood products. The idea that these mechanisms can contribute to the development of new infarcts was recently confirmed in a study of 11 patients with aSAH [22]. In this study, subdural opto-electrodes were used to monitor cortical tissue at risk for development of delayed infarcts. Typically ischemic episodes started with a cluster of repetitive SDs and progressively prolonged spreading ischemias. The SDs eventually became terminal, without recovery, and were followed by a negative ultraslow potential (NUP) that is thought to reflect the progression from persistent depolarization to cell death in an increasing fraction of neurons. Accordingly, NUP-displaying electrodes were significantly more likely to overlie a developing infarction than those not displaying a NUP. During the NUP, the median duration of spreading ischemia was 40 min, CBF fell from 57 to 26%, and tissue partial pressure of oxygen abruptly decreased from 13 to 3 mmHg.

In conclusion, the present case suggests that proximal vasospasm is not a *conditio* sine qua non for the development of delayed infarcts after tSAH, similar to results found previously for aSAH. A multidisciplinary international research group has previously recommended that the presence of angiographic vasospasm should not be a criterion to diagnose delayed infarcts after aSAH [23]. The present results suggest the same for delayed infarcts after tSAH. Although aSAH and tSAH differ in the mechanism of primary injury (aneurysm rupture vs. trauma), we suggest that they may share common mechanisms of secondary injury [24, 25]. Further systematic research is needed on this topic, and for this purpose, we recommend that serial MRI scans should be performed more frequently after tSAH.

## Abbreviations

ADC: apparent diffusion coefficient
aSAH: aneurysmal subarachnoid haemorrhage
CRP: C-reactive protein
CT: computed tomography
CTA: computed tomography angiography
DWI: diffusion weigthed imaging
INR: international normalized ratio
MCA: middle cerebral artery
MRA: magnetic resonance angiography
MRI: magnetic resonance imaging
NUP: negative ultraslow potential
PCA: posterior cerebral artery
SD: spreading depolarization
TCD: transcranial Doppler sonography
tSAH: traumatic subarachnoid hemorrhage

## References

[1] Neil-Dwyer G, Lang DA, Doshi B, Gerber CJ, Smith PW (1994). Delayed cerebral ischaemia: the pathological substrate. Acta Neurochir.

[2] Stoltenburg-Didinger G, Schwarz K, Cervós-Navarro J, Ferszt R (1987). Brain lesions secondary to subarachnoid hemorrhage due to ruptured aneurysms. Stroke and microcirculation.

[3] Schatlo B, Dreier JP, Glasker S, Fathi AR, Moncrief T, Oldfield EH (2010). Report of selective cortical infarcts in the primate clot model of vasospasm after subarachnoid hemorrhage. Neurosurgery.

[4] van Norden AG, van Dijk GW, van Huizen MD, Algra A, Rinkel GJ (2006). Interobserver agreement and predictive value for outcome of two rating scales for the amount of extravasated blood after aneurysmal subarachnoid haemorrhage. J Neurol.

[5] Dreier JP, Sakowitz OW, Harder A, Zimmer C, Dirnagl U, Valdueza JM (2002). Focal laminar cortical MR signal abnormalities after subarachnoid hemorrhage. Ann Neurol.

[6] Woitzik J, Dreier JP, Hecht N, Fiss I, Sandow N, Major S (2012). Delayed cerebral ischemia and spreading depolarization in absence of angiographic vasospasm after subarachnoid hemorrhage. J Cereb Blood Flow Metab.

[7] Weidauer S, Vatter H, Beck J, Raabe A, Lanfermann H, Seifert V (2008). Focal laminar cortical infarcts following aneurysmal subarachnoid haemorrhage. Neuroradiology.

[8] Brown RJ, Kumar A, Dhar R, Sampson TR, Diringer MN (2013). The relationship between delayed infarcts and angiographic vasospasm after aneurysmal subarachnoid hemorrhage. Neurosurgery.

[9] Perrein A, Petry L, Reis A, Baumann A, Mertes P, Audibert G (2015). Cerebral vasospasm after traumatic brain injury: an update. Minerva Anestesiol.

[10] Vora YY, Suarez-Almazor M, Steinke DE, Martin ML, Findlay JM (1999). Role of transcranial Doppler monitoring in the diagnosis of cerebral vasospasm after subarachnoid hemorrhage. Neurosurgery.

[11] Coyne TJ, Loch Macdonald R, Christopher WM (1994). Angiographic vasospasm in a contemporary series of patients with aneurysmal subarachnoid haemorrhage. Journal of clinical neuroscience : official journal of the Neurosurgical Society of Australasia.

[12] Mills JN, Mehta V, Russin J, Amar AP, Rajamohan A, Mack WJ (2013). Advanced imaging modalities in the detection of cerebral vasospasm. Neurol Res Int.

[13] Kerkeni H, Schatlo B, Dan-Ura H, Remonda L, Muroi C, Diepers M (2015). Proximal arterial diameters on CT angiography and digital subtraction angiography correlate both at admission and in the vasospasm period after aneurysmal subarachnoid hemorrhage. Acta Neurochir Suppl.

[14] Ohkuma H, Manabe H, Tanaka M, Suzuki S (2000). Impact of cerebral microcirculatory changes on cerebral blood flow during cerebral vasospasm after aneurysmal subarachnoid hemorrhage. Stroke.

[15] Dreier JP (2011). The role of spreading depression, spreading depolarization and spreading ischemia in neurological disease. Nat Med.

[16] Iwanowski L, Olszewski J (1960). The effects of subarachnoid injections of iron-containing substances on the central nervous system. J Neuropathol Exp Neurol.

[17] Hartings JA, York J, Carroll CP, Hinzman JM, Mahoney E, Krueger B (2017). Subarachnoid blood acutely induces spreading depolarizations and early cortical infarction. Brain.

[18] Dreier JP, Korner K, Ebert N, Gorner A, Rubin I, Back T (1998). Nitric oxide scavenging by hemoglobin or nitric oxide synthase inhibition by N-nitro-L-arginine induces cortical spreading ischemia when K+ is increased in the subarachnoid space. J Cereb Blood Flow Metab.

[19] Dreier JP, Petzold G, Tille K, Lindauer U, Arnold G, Heinemann U (2001). Ischaemia triggered by spreading neuronal activation is inhibited by vasodilators in rats. J Physiol.

[20] Dreier JP, Major S, Manning A, Woitzik J, Drenckhahn C, Steinbrink J (2009). Cortical spreading ischaemia is a novel process involved in ischaemic damage in patients with aneurysmal subarachnoid haemorrhage. Brain.

[21] Hinzman JM, Andaluz N, Shutter LA, Okonkwo DO, Pahl C, Strong AJ (2014). Inverse neurovascular coupling to cortical spreading depolarizations in severe brain trauma. Brain.

[22] Luckl J, Lemale CL, Kola V, Horst V, Khojasteh U, Oliveira-Ferreira AI (2018). The negative ultraslow potential, electrophysiological correlate of infarction in the human cortex. Brain.

[23] Vergouwen MD, Vermeulen M, van Gijn J, Rinkel GJ, Wijdicks EF, Muizelaar JP (2010). Definition of delayed cerebral ischemia after aneurysmal subarachnoid hemorrhage as an outcome event in clinical trials and observational studies: proposal of a multidisciplinary research group. Stroke.

[24] Balanca B, Meiller A, Bezin L, Dreier JP, Marinesco S, Lieutaud T (2017). Altered hypermetabolic response to cortical spreading depolarizations after traumatic brain injury in rats. J Cereb Blood Flow Metab.

[25] Dreier JP, Fabricius M, Ayata C, Sakowitz OW, William Shuttleworth C, Dohmen C (2017). Recording, analysis, and interpretation of spreading depolarizations in neurointensive care: review and recommendations of the COSBID research group. J Cereb Blood Flow Metab.

